# RNA diagnostics and therapeutics: a comprehensive review

**DOI:** 10.1080/15476286.2024.2449277

**Published:** 2025-01-03

**Authors:** Adeela Fathima Saju, Aditi Mukundan, Divyashree MS, Raghu Chandrashekhar, Archana Mahadev Rao

**Affiliations:** a Department of Biotechnology, Manipal Institute of Technology (MIT), Manipal Academy of Higher Education (MAHE), Manipal, India; b Department of Pharmaceutical Biotechnology, Manipal College of Pharmaceutical Sciences (MCOPS), Manipal Academy of Higher Education (MAHE), Manipal, India

**Keywords:** Antisense Oligonucleotide, aptamers CRISPR, mRNA vaccine, PCR, RNA-Seq, RNA therapy and diagnostics

## Abstract

RNA-focused therapy and diagnostics have been making waves in molecular biology due to the advantages RNA has over DNA; for instance, the ability of RNA to target nearly any genetic component in the cell is a big step in treating disorders. Moreover, RNA-based diagnosis of diseases is only becoming increasingly popular, especially after the COVID-19 pandemic, which brought up the need for cost-effective and efficient diagnosing kits for the vast majority. RNA-based techniques also have close to no risk of genotoxicity and can efficiently target undruggable regions of the cell. RNA treatments have effectively shown the future of the medical industry in the past couple of decades, and they will only be seen to improve. This review paper provides an overview on the different techniques that use RNA-based approaches in the field of diagnostics and therapeutics.

## Introduction

The field of therapeutics and diagnostics development has progressed by leaps and bounds in bringing forth a variety of approaches to treat various disease conditions. With the increasing threat of new pathogenic varieties, the necessity for development of new therapeutic and faster diagnostics remains the need of the hour. The use of small molecule drugs has been used in the field of therapeutics for a long time, but the development of resistant varieties and the difficulty of administering the developed formulations have proved a major roadblock in the drug development and diagnostics field. In the field of therapeutics, developing a technique that can detect the presence of the infectious agent well in time will help save the lives of millions. The recent COVID-19 pandemic has been a wake-up call for humankind to be ready for new advancements in the field of therapeutics and diagnostics.

One such approach that has contributed tremendously to advancement in this area is the use of RNA-based techniques. The simplicity of developing sequence-based therapeutics and diagnostics makes the approach very potent and prevents host-related off-target effects. The method for identifying foreign infections should be quick, accurate, sensitive, instrument-free, and economical and RNA-based diagnostics meet the criteria mentioned along with the development of new technologies. For example, PCR has increased the ability to diagnose several diseases by making it possible to identify numerous human infections that were previously challenging to detect because of their low concentration in the sample. It is an efficient, non-intrusive, and extremely precise process. Moreover, with the recent development of aptamers and CRISPR strategy, RNA-based drug and therapeutic designing are gaining impetus and will soon be a popular tactic.

## Brief history of RNA therapeutics and diagnostics

The messenger RNA or mRNA was discovered by Jacob, Monod, Crick, Benner and Meselson and more in 1961, and the central dogma shows how the RNA molecule is the transcript of information that is carried by the DNA. The central dogma depicts that any gene that needs to be expressed should produce an RNA copy that may be further translated to a protein. The RNA is the initial transcript that needs to be expressed for any protein to be synthesized. Using this intermediate molecule, several therapeutics and diagnostics have been designed.

### RNA diagnostics

RNA diagnostics and therapy is a revered field with an immense amount of potential, and it all began in 1868 when the Friedrich Miescher first isolated a new compound from the nuclei of white blood cells, which was later termed nucleic acid [1]. After countless more discoveries and close to a century later, between 1930 and 1950, it was discovered that RNA and DNA have distinct properties. It was also during this period that they came across the fact that RNA and DNA were in different parts of the cell. Primarily, DNA is found in the nucleus in the case of eukaryotes, and due to various forms of RNA, there are specific localizations for each type (mRNA, rRNA and tRNA) [2].

Diagnosis using the RNA of the host began with the discovery of Polymerase Chain Reaction (PCR) in 1985 (first demonstrated), which was later elaborated over the years [3]. PCR, especially for diagnostics, was first developed for the detection of mutations in the HBB gene that causes sickle cell anaemia [4]. After many trials, scientists honed PCR to unveil quantitative PCR (qPCR) and Real time qPCR (RT-qPCR) for definitive analysis. The use of RT-PCR is well known for the diagnosis of viral infections like COVID-19 for comparatively economic and conclusive results.

Microarrays came into the picture in 1995, which was the first cited use of the technology by Patrick Brown and colleagues [5]. In the early microarrays, cDNA clones were used as PCR templates, and the final PCR products served as the probes. A handcrafted robot was utilized to print the array, which was then employed to determine the patterns of gene expression in parallel with 48 genes of *Arabidopsis thaliana*. Microarrays play a key role in diagnosing and treating infectious illnesses as well as discovering novel tumour subtypes and prognostic groupings [6]. Perou and colleagues were the first to categorize breast carcinomas into discrete subtypes using RNA microarrays. These subtypes are designed on the different expression patterns of a subset of genes known as the ‘gene set’ or ‘gene signature’ which was employed for prognosis of 78 individuals with breast cancer in a follow-up analysis [7].

RNA sequencing was developed in the mid-2000s, but the first-generation sequencing, also known as Sanger sequencing, was initiated by Sanger in 1977, employing the chain termination method [8]. This was followed by the chemical degradation method developed by Maxim and Gilbert. Many methods were also developed and flourished for various applications. Some strategies also came about with the help of microarrays, and hence, microarray technology and RNA sequencing are often compared due to the similarities they share with each other. RNA sequencing was first utilized in diagnostics in 2017 [9]. Several studies have carried out RNA-seq on several tissues, like fibroblast, muscle, and blood. The studies used different methods and shed light on the diagnostic application of RNA sequencing.

Diagnosing diseases is essential and a prerequisite for efficient treatments and therapy. Recent developments in diagnostic technologies aim to provide accurate and reasonable tests at home. The above-mentioned methods have been in use for a long time for various applications, but they are not cost efficient and require trained personnel. Therefore, there is a need for technology that combines ease of use and cost-efficiency along with the diagnostic accuracy of PCR. Here comes CRISPR (for Clustered Regularly Interspersed Short Palindromic Repeats)-based diagnostics, which offer the necessary capacity to meet these unmet demands.

### RNA therapeutics

The application of RNA-based compounds for the treatment, elimination and prevention of disease is known as ‘RNA therapy’. The creation of RNA-based treatments for a wide range of uses has been made possible by recent developments in the production, purification, and transport of RNA to cells. RNA-based therapeutics will bring a massive change in how diseases are cured and make tailored medicine easily accessible. They can target previously un-targetable pathways accessible and are also improving ease of manufacture. As research, academic organizations and even small businesses can make these new and customize synthesized RNA structures, which is a multifaceted therapeutic technique [10].

**Figure 1.**
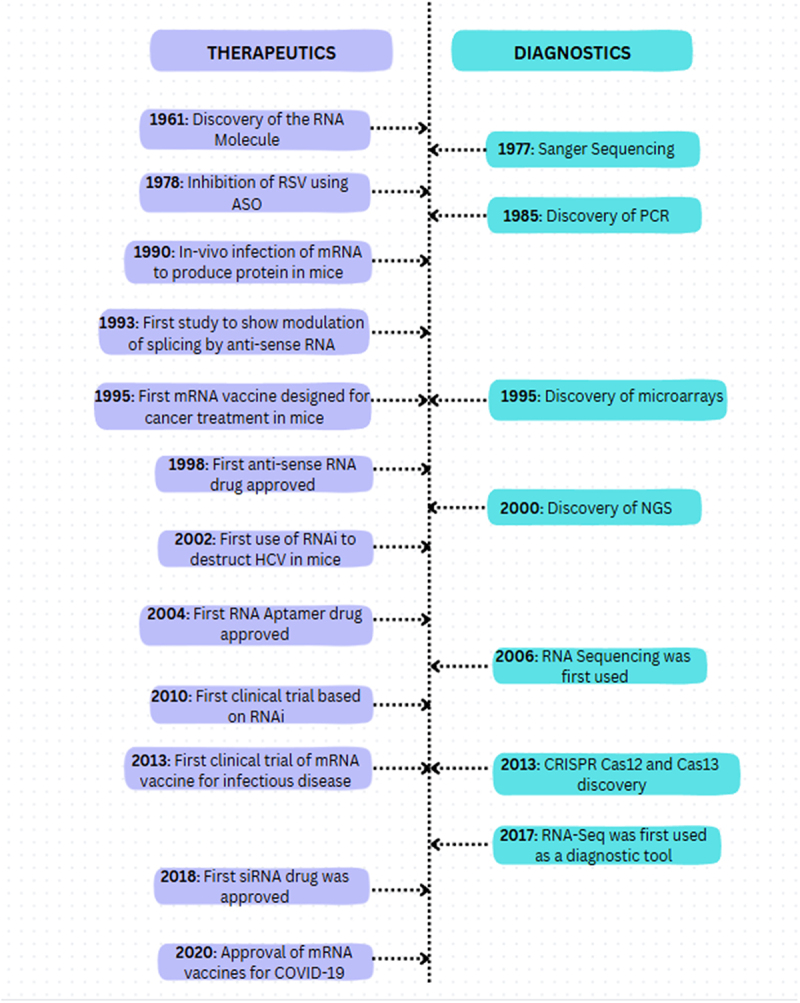
A timeline corresponding to various breakthroughs in RNA diagnostics and therapeutics.

The capacity of complementary pairing between two RNAs with each other was critical in the field of RNA. Figure 1 provides an insight into the timeline of advancements made in RNA-based diagnostics and therapeutics. Earlier RNA researchers had no idea that RNA could create a double stranded structure, even though this knowledge is taken for granted today. However, the first nucleic acid hybridization procedures, published by Rich and Davies in 1956, showed that RNA could develop a structure using the complementarity of its nitrogenous bases [11]. The later discoveries of RNA interference (RNAi) and microRNAs (miRNAs), where the formation of double stranded RNA is important for RNA silencing, were made due to this finding [12]. To stop replication of the virus, Stephenson and Zamecnik used RNA base pairing for the first time in 1978 by the use of antisense oligonucleotide (ASO) that targeted the Rous sarcoma virus’s (RSV) 35S RNA sequence [13].

RNA splicing is the posttranscriptional process of ligating exons and splicing introns after synthesizing the primary RNA transcript. It is almost impossible to restore or amend these splicing anomalies with traditional small molecule-based therapies. However, RNA-based drugs can accomplish this. In a 1993 study, it was initially discovered that antisense oligos might regulate alternative splicing [14]. It was seen that a series of antisense oligonucleotides focussing the branch points and splice sites of the pre-mRNA was employed to rectify the aberrant splicing of the thalassaemic pre-mRNA and ameliorate symptoms.

In 1978, it was found that a 21-nucleotide sequence downstream of the 5’ cap was identical to a 21-nucleotide sequence upstream of the 3’ end for the Rous Sarcoma Virus (RSV) 35S RNA. These sequences had a major function in the circularization of the viral DNA right before it gets integrated into the host cell. The circularization step is vital for the further production of RSV, so to stop its proliferation, it can be put to a halt by introducing a synthesized Anti-Sense Oligonucleotide, which was had base complementarity with the 13-ribonucleotide segment of the reiterated sequences. The ASO binds to the terminal sequence and inhibits the circularization, thus stopping the production of RSV [13].

Gene therapy was introduced in the early 1970s and used to rely on introduction genetic information into tissues indirectly. The target cells were isolated, genetic material was introduced using viral vectors and put back into the body. The direct introduction of new genetic material into the tissues without the involvement of vectors was essential. It was achieved either by encapsulating DNA in liposomes or it was coupled to a polylysine-glycoprotein carrier complex. The in vivo injection of mRNA to produce protein in mice was first attempted in 1990, and the direct injection of RNA and DNA into the muscle cells of mice resulted in the expression of the reporter genes [15].

Antisense oligonucleotides typically work by hybridizing with the targeted RNA, which either causes it to be degraded by cellular RNase H or causes a stop in its translation. In a different strategy, so-called antigene oligonucleotides are used to target certain DNA regions, where they create triplex structures and prevent RNA polymerase-II from starting transcription. The antisense and antigene oligonucleotides lead to the downregulation of targeted genes and are used to bring down the intracellular level of undesirable products produced by viruses, other pathogens, or oncogenes. ASOs were used to restore the aberrant splicing of mutated β-globin pre-mRNAs. The mutations that were causing the aberrant splicing were found to be the underlying cause of β-thalassaemia. Thus, the correct splicing was restored in thalassaemic pre-mRNA by ASOs [14].

In the field of cancer treatment, the first mRNA vaccine came into the picture in 1995. mRNA transcripts that translate the human carcinoembryonic antigen (CEA) and luciferase were constructed. They induced expression of luciferase *in vivo* following intramuscular injection in mice. The mRNA-immunized mice showed an anti-CEA antibody response, and the control mice showed none. This study expanded new horizons for cancer treatment [16].

The first antisense RNA drug – Fomivirsen (Vitravene) – was approved by the FDA for the treatment of CMV (cytomegalovirus) retinitis in 1998. It was administered by injecting it into the eye monthly. It was useful for patients who were resistant to other treatments of CMV [17].

RNAi, or RNA interference, was first characterized in 1998 in *Caenorhabditis elegans* embryos by using sense and anti-sense RNA for treatment. This brought about the sensitive and potent blocking in the expression of targeted mRNAs. As this strategy is straightforward and effective, it was used widely in a short amount of time. RNAi has been used to prevent the reproduction of the hepatitis C virus in mice. This led to a broad examination of RNAi for therapeutic purposes [18]. Sequence-specific silence of homologous genes is how the evolutionarily conserved surveillance mechanism known as RNA interference (RNAi) reacts to double-stranded RNA. It was demonstrated that small-hairpin RNAs produced in vivo from DNA templates and synthetic small interfering RNAs can both reduce transgene expression in adult mice. The therapeutic potential was also shown by demonstrating efficient targeting of a hepatitis C virus by RNAi in vivo [19]. This experiment with RNAi permitted the first RNAi-based clinical studies in 2010, where a patient with extensive melanoma was treated with a siRNA that targets the M2 subunit of ribonucleotide reductase. In this trial, the siRNA was successfully delivered in a targeted nanoparticle to the mRNA. After this trial, many siRNA-based drugs were tested for various diseases, and in 2018, the first siRNA drug for patients having transthyretin-mediated amyloidosis was approved [20].

The first RNA Aptamer drug – Pegaptanib – was the first RNA aptamer drug approved for the treatment of neovascular age-related macular degeneration in 2004. Treatment with pegaptanib decreased the possibility of the study eye developing legal blindness and improved vision stability. This drug validated the usage of Aptamer-based therapy [21].

In 2013, researchers launched the first clinical study for mRNA vaccination against an infectious illness, intending to assess the effectiveness of a new rabies vaccine that delivers an mRNA coding for the disease’s glycoprotein. The analyses showed that vaccination produced functional antibodies specific for the viral antigens, providing a resounding validation for the method. Due to mRNA’s non-infectious and non-integrative properties in the human body, mRNA vaccines are considered superior to traditional immunizations. Yet, there were challenges in drawing up conclusive results due to the unstable nature of mRNA. In 2020, most governments approved the mRNA-based vaccine for severe acute respiratory syndrome coronavirus 2 (SARS-CoV-2). This concise overview highlights the long history of RNA therapies. It reveals how recent developments in mRNA vaccines were made possible by the findings of several investigations carried out for more than four decades.

Patisiran (Onpattro) was the first siRNA drug to be approved in 2018 to treat transthyretin amyloidosis. Patients with familial transthyretin amyloidosis were shown to have considerably better neuropathy after receiving the RNAi therapy Patisiran. The effects persisted across patient subgroups and included the sensorimotor and autonomic areas. Additionally, Patisiran therapy produced notable increases in quality of life, walking, nutritional status, and daily living activities [18]. Following the approval of Patisiran in 2018, several siRNA-based therapeutics have been approved for therapy like Givosiran (Givlaari), Lumasiran (Oxlumo), Inclisiran (Leqvio), Vutisiran (Amvuttra) and Nedosiran (Rivfloza). They have been approved for the treatment of conditions ranging from acute hepatic porphyria to amyloidosis neuropathy [22].

## RNA diagnostic techniques

### RNA sequencing (RNA-seq)

RNA-seq has greatly improved diagnostic yield in genetically unresolved cases of Mendelian diseases. With Whole Genome Sequencing (WGS), DNA is isolated, sequenced by De Novo Sequencing and other methods and analysed to check similarity to other sequences and information about diseases. With whole Exome Sequencing (WES): the exome is sequenced and only 50% of patients were receiving a molecular diagnosis. There was a high fraction of Variants with Uncertain Significance (VUS) and challenges of interpretation. To overcome these challenges, RNA-seq was implemented. It is a minimally invasive procedure. It can even reveal extreme cases of mono-allelic expression (MAE) [23]. About 50–75% of the patients do not receive a genetic diagnosis through WES/Whole Exome Sequencing and WGS/Whole Genome Sequencing. This is where RNA Sequencing comes into play [24].

**Figure 2.**
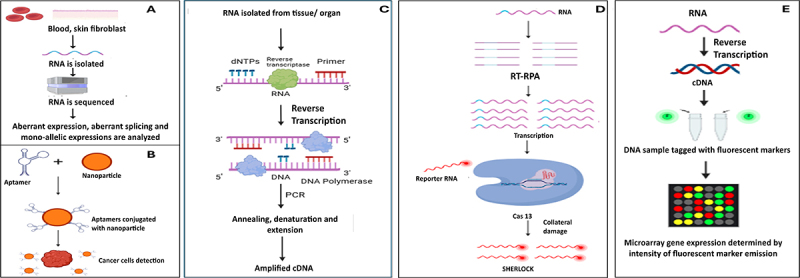
Flowcharts depicting A. RNA-Seq mechanism, B. Cancer diagnosis using RNA aptamers, C. RT-PCR mechanism, D. CRISPR-Cas13 detection system, E. Diagnosis using microarray.

The procedure for RNA seq is depicted in Figure 2. RNA is collected and isolated from a tissue source, typically from blood and skin fibroblasts. The tissue chosen is vital because not every gene is expressed in all tissues and not all tissues are easily reachable. Reverse transcription is used in the following step to transform RNA fragments (usually mRNA) into complementary DNA (cDNA), creating a cDNA library. Following NGS sequencing of this sample of cDNA, often using Illumina short-read sequencing, millions of reads are generated, which are then aligned to a reference genome (UCSC Genome Browser) for further downstream analysis. RNA-seq offers the capacity to identify aberrant gene expression levels: expression levels are quantified by counting how many reads are mapped to each gene. This value can be used to identify expression outliers that might be clinically significant by comparing it to a control data set; variations in gene splicing, allele-specific expression (ASE): the expression of only one allele, while the other is silenced; and other transcriptome data, in contrast to DNA sequencing, which concentrates solely on the nucleotide sequences. RNA-Seq increased the diagnostic rate by 7.5% to 36%, which is more than WGS and WES [24,25].

### RNA aptamers

RNA aptamers are comparable to antibodies – they are made to bind to specific molecules. They are generated *in vitro* using a procedure called SELEX for a distinct function. Their high specificity and affinity allow for it to function as a diagnostic tool [26,27].

Methods like immunohistochemistry, flow cytometry and MRI are employed in the detection of cancer, but they are time-consuming and cannot detect a low load of cancer cells. Aptamers, on the other hand, can detect as low as 10 cancer cells. Aptamers can even identify metabolic products produced by cancer cells and cancer biomarkers. The commonly used system for cancer diagnosis using aptamers is when they are conjugated with nanoparticles. These conjugates can recognize cancer cells in bodily fluids like serum and blood. Tumour cell-specific aptamers can be immobilized on the nanoparticles’ surface. The aptamers perform the specific detection of the cancer cells, and the nanoparticles prevent the degradation of the aptamers. Thus, the detection of the cells helps in diagnosing the cancer (Figure 2) [28,29].

### qRT-PCR

Polymerase Chain Reaction (PCR) has been used to amplify DNA sequences and has changed the landscape of molecular biology research. Its expansion to RNA sequences has been achieved by using the reverse transcriptase enzyme to make cDNA, which is further amplified by PCR. This method has been coined as Reverse Transcription Polymerase Chain Reaction (RT-PCR) [30]. RT-PCR also stands for Real Time Polymerase Chain Reaction – the conventional PCR method was enhanced such that the quantifying process could be made easier and could be observed in real-time. It gained worldwide recognition due to the recent COVID-19 pandemic [31]. RT-PCR provides a good mathematical relationship between the sequences before and after amplification because there are only a certain number of replications the sequences are going through. If there is less starting material, the output will also be less, the same holds true for if there was more starting material [32].

RNA is collected from the tissue or organ; for example, swabs were taken from the nose.

The RNA is then converted to cDNA using reverse transcriptase, dNTPs, and relevant primer. The cDNA is then denatured. The template DNA (single strand) is then replicated using forward and reverse primers (annealing), dNTPs, and DNA polymerase. There are several cycles of this replication; this step is known as extension. The result of this amplification can be thousands to millions of copies (Figure 2) [33].

### CRISPR-Cas system

CRISPR is a recent, up-and-coming gene editing technology that revolutionized the field of molecular biology. How it works is simple: CRISPR has a unique way of finding a specific part of DNA in the cell, after which the protein and the gDNA or gRNA are allowed to alter the specific part of the DNA. CRISPR technology also has the potential to transform medicine, enabling us to not only treat but also diagnose various diseases. In such an instance, CRISPR-Cas9 has become a powerful tool with great potential to treat diseases. Similarly, Cas13 can also be used to detect disease. First, it hunts for viral DNA using a guide DNA. When it finds its viral gene, Cas13 gets activated, and in some circumstances, it cuts any RNA it encounters; this process is called collateral activity. SHERLOCK™ one of the highly sensitive tools that uses the above process to detect diseases. It is based on the detection of the fluorescent signal detection. Many versions exist wherein the detection is based on the sensing of electrical, electrochemical, or colorimetric signal sensing [34].

Figure 2 gives a quick overview for the detection of low levels of viral RNA present in a sample using the SHERLOCK™ technique. The sample is taken from an affected patient with a possible viral infection. Then, the RNA is amplified using RPA (recombinase polymerase amplification) then add reporter molecules that are sensitive to Cas13. An engineered CRISPR-Cas13 is added to the sample (which detects the viral RNA). The engineered CRISPR is designed with a guide RNA and only binds to the viral RNA. When this occurs, it randomly slices through RNA molecules around it along with reporter molecules. Two sides of the reporter molecule carry a different label, and when it is cleaved, creating a system whereby it can be detected whether the disease is present or not by a commercial flow detection system. If the result is positive, the ends of the reporter molecule collect at a site in the detection system and vice-versa when it is negative [35,36].

CRISPR-based systems are rapid and cheap, and they can identify drug-resistance genes [37]. It can make highly targeted modifications. They are highly specific, and there is a heightened precision in gene editing, even for very small sequences. The CRISPR systems are ‘simple’; there is the requirement of only a crRNA guide sequence, Cas9 nuclease protein and a tracrRNA. They can also be modified against new pathogens or even target resistance causing pathways. They have improved the speed and precision of identifying viral and bacterial nucleic acids (Figure 2).

### RNA microarrays

Microarrays are used to analyse the gene expression levels of multiple genes simultaneously in a particular organism’s genome. The first microarray was made using cDNA, which was a product of PCR [5]. RNA Microarrays are used to measure RNA levels. They have been used to observe and detect regulated genes and pathways. Microarrays are commonly used to determine gene regulation associated with diseases, gene biomarkers of several diseases and gene expression levels or patterns [38]. Microarrays play a vital role in detecting new types of cancer tumours. RNA Microarrays were first used to identify the different subtypes of breast cancer based on the gene expression patterns [8]. This was followed up by a study where the gene signature of the different subtypes were used to forecast the breast cancer in 78 patients [39].

The mRNAs that are to be analysed are reverse transcribed. They can be amplified using PCR if the amount is less. If mRNAs from two different samples are analysed, they will be tagged with different fluorescent markers. They are mixed and then hybridized to the microarray. The extra fluorescent markers are washed off. The intensity of each marker is measured using a laser scanner. The intensity is proportional to the number of genes expressed [40].

## RNA based therapeutics

### Antisense oligonucleotides

Antisense RNAs have displayed the property of sequence complementarity to the target molecules, like mRNAs. They bind to it and modify protein expression. They can modify the splicing of pre-mRNAs, influence the deterioration of target mRNAs through RNase H-mediated degradation or block translation into proteins [41].

**Figure 3.**
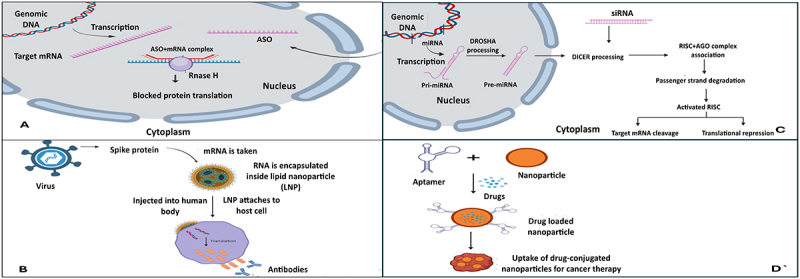
Mechanisms of RNA based therapeutics is depicted in the given image. A. Antisense oligonucleotide, B. miRNA and siRNA mechanisms, C. mRNA vaccine mechanism, D. RNA aptamers for therapy.

One type of ASO is RNase H dependent (Example: Mipomersen, FDA approved in 2013) and other is RNase H independent/steric block (Example: Eteplirsen, FDA approved in 2016). RNAse H dependent method is widely used and is reliant on the RNase H enzyme that breaks down the target RNA. They are better at gene knockdown than RNase H-independent ASOs. Steric block ASOs inhibit translation or splicing. They can prevent polyadenylation, inhibit, or enhance translation or modify splicing (Figure 3) [10].

The mechanism varies depending on the target. There are four types – RNA knockdown, splice modulation, steric translation inhibition and modulation of translation. For the knockdown of RNA – ASOs have small fragments of DNA that bind to the target mRNA or pre-mRNA. This duplex is identified by RNase H, and the phosphodiester bonds are cleaved; thus, the target molecules are destroyed, and no protein synthesis will take place [42]. Splice modulation is the process by which splice enhancers or repressors are blocked by the binding of ASOs to pre-mRNA near exonic regions. This gives rise to a different kind of splicing and can prevent mutations [43]. ASOs inhibit steric translation by binding directly to the mRNA region that is close to a start codon [44]. ASOs can enhance protein expression by translation modulation. This is done by the binding of the ASO to the 5’ untranslated region (UTR) upstream of the pertinent exon, reducing the attachment of the ribosomes to it and, in turn, increasing the binding of the ribosomes to the start codon [45].

### siRNAs

Small interfering RNA (siRNA) is small, non-coding, double-stranded RNA used in gene regulation. They do so by cleaving mRNA. They are efficient in gene knockdown. siRNAs regulate the same genes that express them.

The DICER (specialized ribonuclease III-like enzyme) converts dsRNA, which can be intentionally injected or transcribed, into siRNA, which is then inserted into the RNA-induced silencing complex (RISC). The RISC enzyme AGO2 (endonuclease argonaute 2) cleaves the passenger strand, or sense strand, of siRNA. The guide strand (antisense strand) directs the active RISC towards the target mRNA. The breakdown of mRNA occurs when the guide strand of siRNA fully complements the target mRNA (Figure 3) [41,46]. In 2018 and 2019, the FDA authorized Patisiran and Givosiran, two siRNA medications. Hereditary transthyretin-mediated amyloidosis is treated with Patisiran. It cleaves the mRNA by attaching itself to the 3′ UTR. Givosiran is used to treat acute hepatic porphyria. It attaches itself to the mRNA of delta-aminolevulinic acid synthase 1 (ALAS1) and inhibits its translation, which lowers the neurotoxic intermediates in this malady [1,47].

### miRNA

microRNAs are single-stranded RNA that regulate the expression of multiple mRNAs at the same time. They do so by promoting the degradation of target mRNAs or by inhibiting translation. miRNAs are expressed by genes whose function is to make miRNAs, but those mRNAs can regulate genes other than the ones that express them.

RNA polymerase II uses the miRNA gene to give rise to pri-miRNA, DROSHA cleaves the pri-miRNA to pre-miRNA. It is carried by exportin five into cytoplasm and is converted by into miRNA by DICER. After the miRNA complexes with the RISC and the passenger strand is removed, the miRNA-RISC complex is partly complementarily bound to the target mRNA by the guide strand. Through translational repression, degradation, or cleavage, the target mRNA is inhibited [48]. There are clinical trials in process for miRNA therapy, none of which have reached the commercial markets yet.

### RNA aptamers

RNA aptamers are single-stranded, 25–80 base pairs long, and are in the form of hairpin structures to facilitate the specific binding to surfaces. They can be used either as ‘antagonists’-those that inhibit a specific protein function by binding tightly to it or ‘agonists’ – for receptors that prevent disease [49]. They are more commonly used as antagonists [27]. The process of these molecules to act as therapeutics is depicted in Figure 3. An RNA Aptamer-Pegabtanib, has been used to treat age-related macular degeneration. It targets the VEGF protein and binds specifically to the 165 isoform and blocks the function [41]. RNA aptamers are also used as delivery systems for siRNAs, small molecule drugs and proteins [27].

The aptamers work by binding to the target protein and modifying or inhibiting its function. It has a high sensitivity to the target molecule, just like an antibody to its antigen. Aptamers’ additional enhancements include reduced costs and better transport into the cells [41].

### Vaccines

A fragment of mRNA that corresponds to a viral protein is introduced by mRNA vaccinations. Cells can create the viral protein by means of this mRNA by utilizing the host protein synthesis machinery. The immune system creates antibodies in response to the foreign protein, so that they can bind to them, and tag the pathogens for elimination. The antibodies will remain in the body, and on second exposure by the same antigen the immune system can respond swiftly. Therefore, if a person is exposed to the same virus that they have been vaccinated against using the mRNA vaccine, the antibodies recognize it, attach to it, and mark it for destruction before it can cause serious illness. (Figure 3)

Regarding messenger RNA vaccines’ mode of action against COVID-19, they supply the genetic code of the pathogen’s relevant antigen. The host then uses this messenger RNA to convert it into the pertinent pathogen protein under study. In other words, the vaccine gives the cells the instructions they need to make the protein. The produced foreign protein can be attacked by the host’s immune system thanks to this mechanism. The injected mRNA will be discarded following the development of the protein. The mRNA has a short life and remains in human tissues for only a few days. The mRNA vaccine will cause the body to mount an immune response without the necessity to endure the actual exposure to the pathogen [50].

### Pros and cons of the RNA-based diagnostics and therapeutics

**Figure 4.**
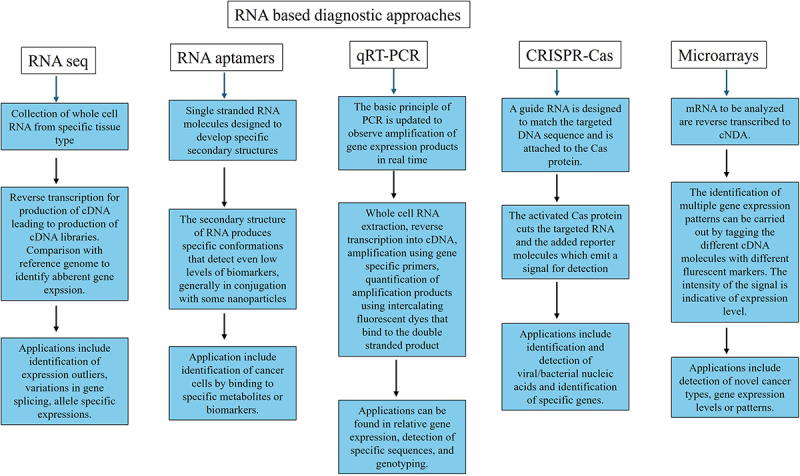
Summary of the RNA based diagnostic approaches. The principle, procedure and application of the different RNA based diagnostic approaches is detailed in the representation.

**Figure 5.**
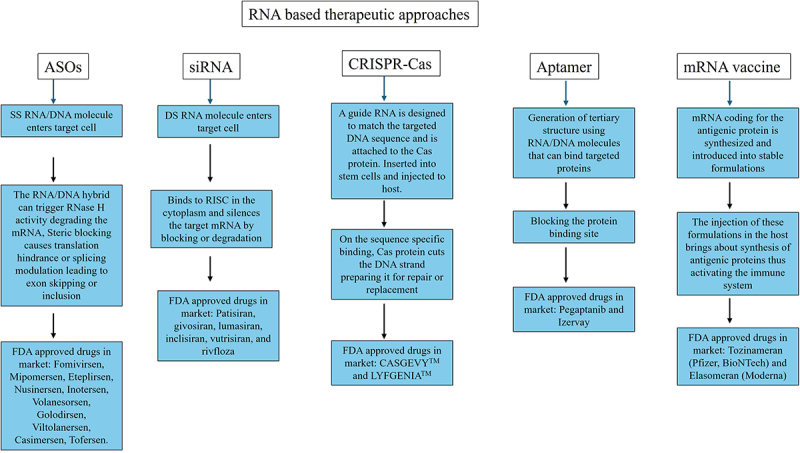
Summary of the RNA based therapeutic approaches. The principle, procedure and FDA approved therapeutics is detailed in the representation.

The different methods offered for the diagnostic and therapeutic applications is a testament to the advancement in the technology. The use of new technology will only keep adding to the efficiency and accuracy of these techniques. The summary of the RNA-based diagnostic and therapeutic approaches is given in Figures 4 and 5.

Aptamers have several advantages to offer in terms of the high thermostability and ability to regenerate even after complete unfolding [51]. Their small size allows them better penetration through the membrane barrier in the vivo setup [52]. They have the huge advantage of producing their activity without any immune activation [53] However, there are several disadvantages also associated with the use of aptamers like the possibility of degradation by nuclease action. This can be overcome by suitable modification of the aptamer sequence [54]. Further, the modification techniques can also help in producing a variety of aptamers against different targets [55]. The qRT-PCR technique offers several advantages like providing sensitive and specific detection of the target gene and providing the result in few hours. The disadvantage of this technique is the necessity of target gene sequence information and the high dependency on the primer efficiency for producing accurate results [56]. The CRISPR-Cas system provides a fast, flexible and cost-effective way for gene editing. However, even this technique faces the delivery limitations and probability of off-target effects [57,58]. Similarly, RNA microarray technique has several advantages like cost-effective and well-established technique. But this technique suffers from drawbacks like high variance for genes that are expressed at low levels and high dependency on the designed probes. Overall, the RNA seq technique is preferred over the microarray technique due to the higher efficiency of the former method [59].

Similarly, in the field of RNA-based therapeutics there are several strategies each of which has its own advantages and disadvantages. For the antisense oligonucleotides, siRNA and miRNA-based therapeutics, the major advantage is reduced off target effects, capacity to target undruggable targets and easy production of variants. However, these strategies also suffer from poor delivery, low concentrations of the therapeutic at the site of action, and poor stability. The development of suitable formulations and specific modifications has drastically helped in overcoming this issue [60,61]. The mRNA vaccine technique has the advantage of being rapid, scalable, and has no requirement for infectious particle for administration. The concerns are raised during the use of mRNA vaccine if they are unstable or give rise to heightened immune response due to non-specific activation [62].

Therefore, each technique that has been introduced for the therapy or diagnostic purposes using the RNA-based method has some pros and cons. The selection of the technique therefore depends on the sample to be analysed, the analysis to be carried out and the available resources with the investigators.

## Conclusion and future prospects

RNA-based treatments were not as widely known before 2020; they became more prominent with the onset of mRNA vaccines for the COVID-19 pandemic. mRNA vaccines were the first ones to be approved against SARS-CoV-2 and this proved useful in acceptance of therapeutics that used mRNA. However, the application of RNA is more than being used as vaccines; it plays a key role in diagnostics, therapeutics, and research. Along with the exponential growth of mRNA vaccines, CRISPR has also been gaining fast attention due to its versatility and functionality. As discussed above, CRISPR is not only used for diagnostics but are actively pursued in other fields, including agricultural research, bioremediation and much more. In addition, some techniques like in situ hybridization are well-established techniques which are used for visualizing the location and expression level of a specific mRNA within a tissue sample. Over the years, this technique has been refined and developed further by using the technological advances for applications in the field of research and diagnostics [63].

The field of RNA-based prognostics, diagnostics and therapeutics is predicted to advance exponentially in the upcoming years. Allied Market Research has predicted approximately $1.2 billion was spent on RNA diagnostics by 2020, and the investments in these fields are expected to reach $6.8 billion by 2028 [64]. The development of novel RNA-based techniques has pushed the technological advancements as well as is evident from various new discoveries like the use of novel biotechnology to produce RNAi agents. This novel technology has created two genres to produce RNA molecules: in vivo by direct expression and using stable carriers in bacterial cells. This circumvents any concerns regarding the effect on physical and chemical properties of the RNA molecules as compared to those produced by chemical or biochemical synthesis [65].

The public has gained access to several RNA-based approaches which have helped in providing an improved and quicker remedy for several issues. Casimersen (Amondys 45), which is an ASO-based therapeutic, is used to treat Duchenne Muscular Dystrophy (DMD), which Sarepta developed after it gained approval from the FDA in 2021 [66]. At the beginning of 2020, the first ever *in vivo* CRISPR gene therapy trial in human patients was run. In the study, paediatric patients with a congenital retinal disease called Leber congenital amaurosis received a CRISPR-based therapeutic system to correct the single base pair mutation causing the disease and be delivered to the retina via local injection of an engineered adenovirus vector. The field of CRISPR therapeutics is ever-growing, from the first CRISPR gene-editing system in human cells in 2013 to the first phase 1 clinical trial in 2020 [36].

ColoSense is an FDA approved multitarget stool RNA test which is non-invasive and used for detection of colorectal cancer and advanced adenomas [67]. RNA diagnostic tools have been used continuously for several infectious diseases; for example, recently, influenza virus RNA has been directly discovered in respiratory secretions using metagenomic RNA-seq, and in a subset of cases, other viral infections have also been found [68]. During the 2014 outbreak in West Africa, RNA-seq was also used to track the Ebola virus’s origin and patterns of transmission [69]. Today, there are multiple FDA approved RNA-based kits that can be used for easy diagnosis of multiple conditions and disorders [70].

In conclusion, RNA-based approaches have played a significant role in tackling emerging and persistent health challenges that appear worldwide. These technologies have offered solutions that are precise, scalable and adaptable. From helping the world in times of global health crisis like COVID-19 by offering vaccines to addressing the ever-present problems like genetic disorders and cancers, RNA-based approaches have demonstrated their immense potential. At the current pace of its advancements, RNA-based technologies are expected to address several unmet medical needs and improve the diagnostic outcomes.

## Data Availability

The data for the information in the paper was obtained from references mentioned in the reference section. No repositories or datasets were used as a reference.

## List of abbreviations

RNA: Ribose nucleic acid
mRNA: Messenger RNA
DNA: Deoxyribose nucleic acid
cDNA: Complementary DNA
COVID: Coronavirus Disease of 2019
PCR: Polymerase Chain Reaction
CRISPR: Clustered Regularly Interspersed Short Palindromic Repeats
RNAi: RNA interference
miRNA: microRNA
ASO: Antisense oligonucleotide
RSV: Rous sarcoma virus
CEA: Carcinoembryonic antigen
CMV: Cytomegalovirus
SARS-CoV-2: Severe acute respiratory syndrome coronavirus 2
qPCR: Quantitative Polymerase Chain Reaction
RT-qPCR: Real time Quantitative Polymerase Chain Reaction
WGS: Whole Genome Sequencing
WES: Whole Exome Sequencing
VUS: Variants with Uncertain Significance
MAE: mono-allelic expression
ASE: Allele-specific expression
dNTP: Deoxy nucleotide triphosphate
UTR: untranslated region
AGO: Argonaute
RISC: RNA induced silencing complex
ALAS1: aminolevulinic acid synthase 1
DROSHA: Double-Stranded RNA-Specific Endoribonuclease
